# P-769. Utility of Urinary Interleukin-6 to aid in the Diagnosis and to assess Prognosis in Urinary Tract Infections

**DOI:** 10.1093/ofid/ofaf695.980

**Published:** 2026-01-11

**Authors:** Anupam Dey, Soumya Kanti Mandal, Debapriya Bandyopadhyay, Debananda Sahoo, Ashoka Mahapatra

**Affiliations:** All India Institute of Medical Sciences, Bhubaneswar, Bhubaneswar, Orissa, India; All India Institute of Medical Sciences, Bhubaneswar, Bhubaneswar, Orissa, India; All India Institute of Medical Sciences, Bhubaneswar, Bhubaneswar, Orissa, India; All India Institute of Medical Sciences, Bhubaneswar, Bhubaneswar, Orissa, India; All India Institute of Medical Sciences, Bhubaneswar, Bhubaneswar, Orissa, India

## Abstract

**Background:**

Urinary tract infections (UTIs) are among the most common infections encountered globally. Diagnosis often relies on symptoms, pyuria, and urine cultures. However, traditional methods like urine culture are time-consuming and lack immediate applicability. This study evaluates the role of urinary IL-6 as a diagnostic and prognostic biomarker, exploring its potential to guide treatment and augment antimicrobial stewardship practices.

**Fig 1.**
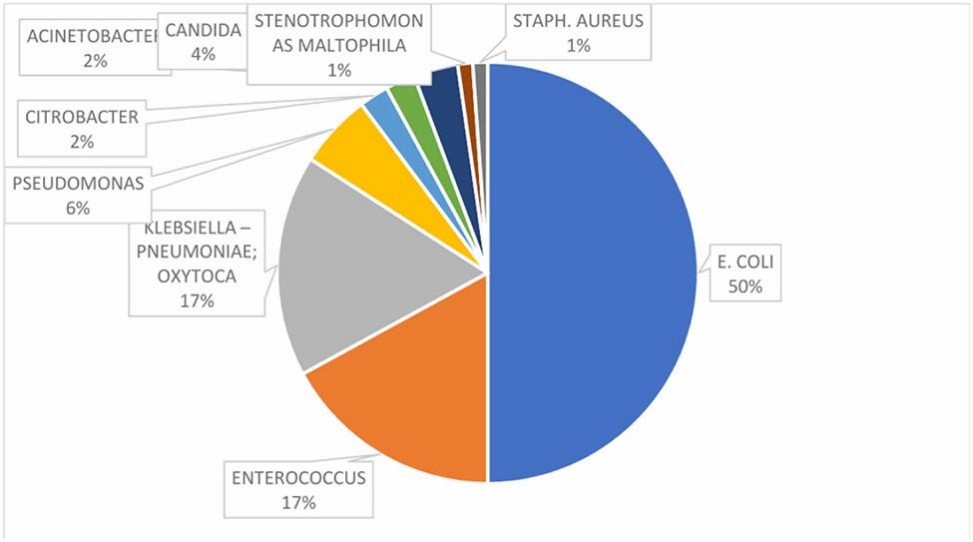
Distribution pattern of isolated organisms

**Fig 2.**
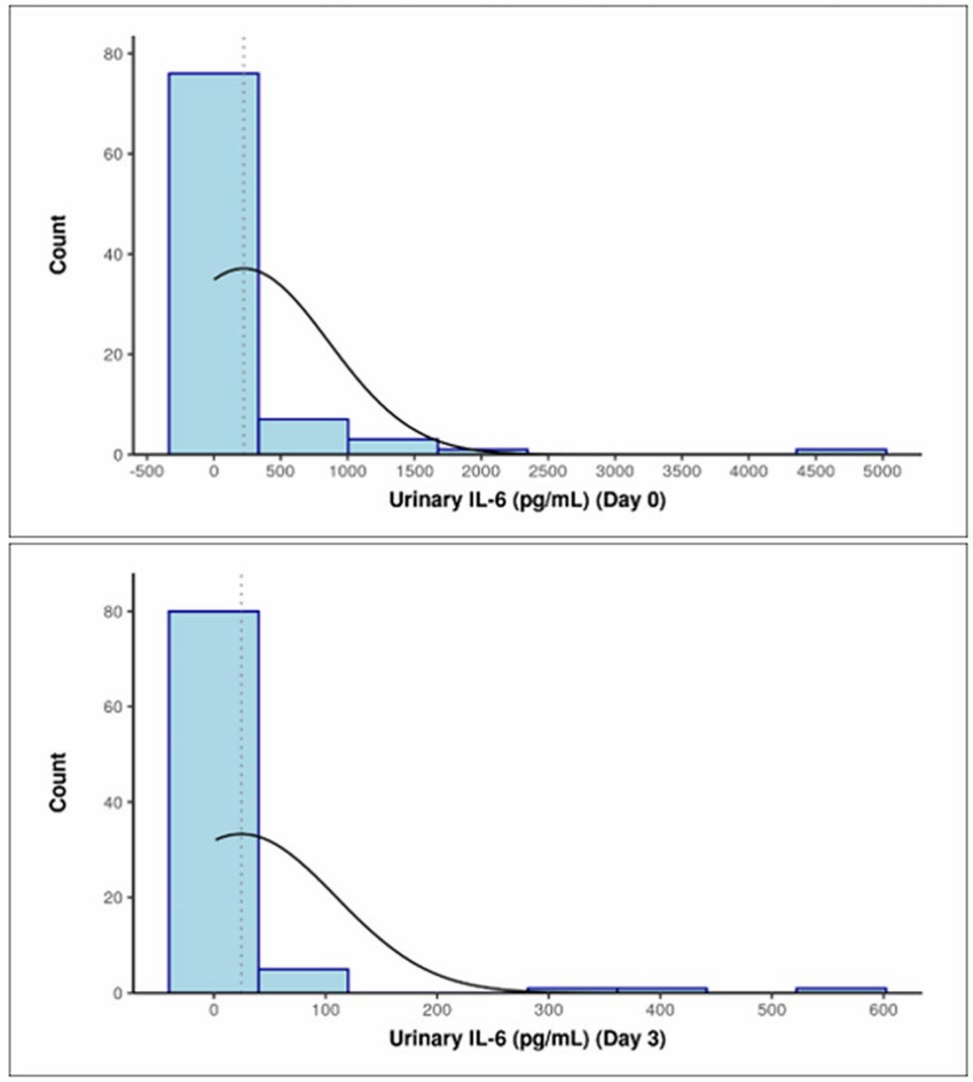
Distribution of Urinary IL-6 on day 0 and day 3

**Methods:**

This hospital-based observational study enrolled 88 patients aged >15 years with culture-positive UTIs. Urinary IL-6 levels were assessed before and 72 hours after antibiotic initiation. IL-6 was analysed using the Roche Elecsys® e801 automated immunoassay analyser. Clinical data, including symptoms, laboratory markers (WBC counts, urine pus cells, urine culture), and antibiotic use, were recorded. Data were analysed using SPSS v28. IL-6 cutoffs were evaluated using receiver operating characteristic (ROC) curves. Statistical significance was set at p < 0.05.

**Fig 3.**
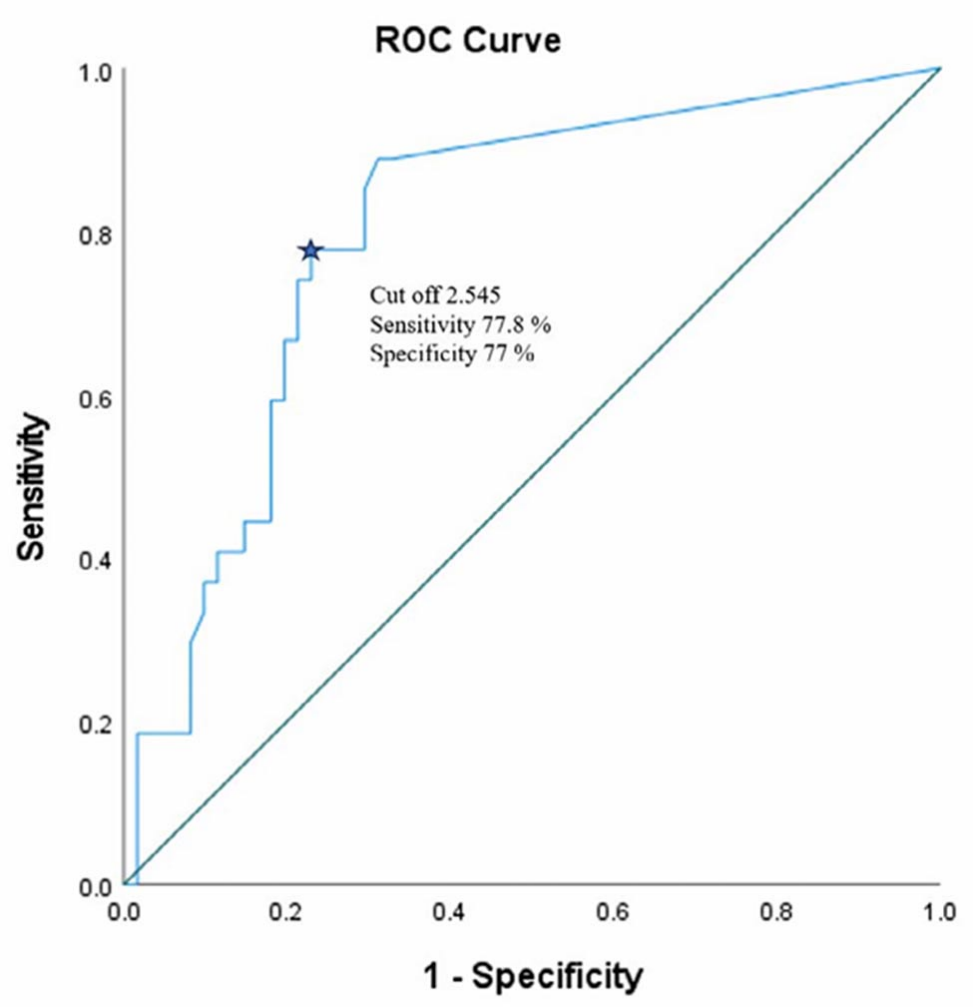
Sensitivity and Specificity of IL-6 on day 3 predicting active infection

**Fig 4.**
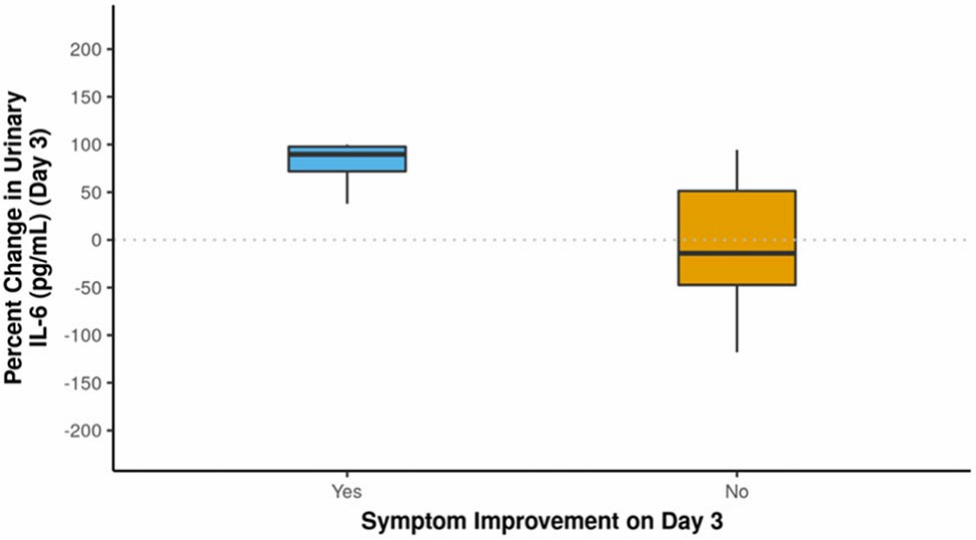
Association Between Symptom Improvement on Day 3 and Percent Change in Urinary IL-6 (pg/mL)

**Results:**

The study population had an average age of 50.26 years, with a female predominance (62.5%). The most common symptom was fever (87.5%), followed by dysuria (73.9%). E. coli was the leading pathogen (50%), followed by Klebsiella and Enterococcus (17% each) (Fig 1). Piperacillin-tazobactam was the most frequently prescribed antibiotic. Mean antibiotic duration was 9.35 days. Urinary IL-6 levels on Day 0 were significantly elevated in UTI cases (mean 223.46 pg/mL), and decreased significantly after 72 hours of antibiotic therapy (mean 24.57 pg/mL) (p< 0.001) (Fig 2). IL-6 levels correlated with WBC counts and pus cell counts, indicating its utility in monitoring infection severity. On Day 3, an IL-6 cutoff of 2.545 pg/mL showed 77.8% sensitivity and 77 % specificity for predicting active infection (Fig 3). The percentage reduction in IL-6 levels was strongly associated with symptom improvement (Fig 4).

**Conclusion:**

Urinary IL-6 is a reliable biomarker for assessing UTI severity, treatment response, and infection prognosis. IL-6 levels assessed on day 3 can aid in timely de-escalation or modification of antibiotic therapy. It can complement traditional diagnostic methods and support antimicrobial stewardship practices.

**Disclosures:**

All Authors: No reported disclosures

